# A lipo-polymeric hybrid nanosystem with metal enhanced fluorescence for targeted imaging of metastatic breast cancer

**DOI:** 10.7150/ntno.92410

**Published:** 2024-02-17

**Authors:** Tejaswini Appidi, Rajalakshmi P Sivasankaran, Shubham A. Chinchulkar, Paloma Patra, Kavipriya Murugaiyan, Bantal Veeresh, Aravind Kumar Rengan

**Affiliations:** 1 Dept. of Biomedical Engineering, Indian Institute of Technology Hyderabad, India.; 2 G. Pulla Reddy College of Pharmacy, Hyderabad, India.

**Keywords:** plasmon-enhanced fluorescence, EPR effect, imaging, passive targeting, lipo-polymeric NPs

## Abstract

Cancer metastasis plays a major role in failure of therapeutic avenues against cancer. Owing to metastasis, nearly 70-80% of stage IV breast cancer patients lose their lives. Nanodrug delivery systems are playing a critical role in the therapy of metastatic cancer in the recent times. This paper reports the enhanced permeation and retention (EPR) based targeting of metastatic breast cancer using a novel nano lipo-polymeric system (PIR-Au NPs). The PIR-Au NPs demonstrated an increase in fluorescence by virtue of surface coating with gold, owing to the metal enhanced fluorescence phenomenon as reported in our earlier reports. Enhanced fluorescence of PIR-Au NPs was observed in murine mammary carcinoma cell line (4T1), as compared to free IR780 or IR780 loaded nanosystems (P-IR NPs), when incubated for same time at same concentrations, indicating its potential application for imaging and an enhanced bioavailability of IR780. Significant cell death was noted with photothermal mediated cytotoxicity *in-vitro* against breast cancer cells (MCF-7 and 4T1). An enhanced fluorescence was observed in the zebra fish embryos incubated with PIR-Au NPs. The enhanced permeation and retention (EPR) effect was seen with PIR-Au NPs *in-vivo*. A strong fluorescent signal was recorded in mice injected with PIR-Au NPs. The tumor tissue collected after 72 h, clearly showed a greater fluorescence as compared to other groups, indicating the plasmon enhanced fluorescence. We also demonstrated the EPR-based targeting of the PIR-Au NPs *in-vivo* by means of photothermal heat*.* This lipo-polymeric hybrid nanosystem could therefore be successfully applied for image-guided, passive-targeting to achieve maximum therapeutic benefits.

## Introduction

Cancer ranks among the top contributors of death, with an estimation of 19.2 million new incidences and 9.9 million deaths as reported in 2020 alone^1^. Although, the development of safe and effective cancer therapeutics is perhaps the major area of research in today's era, certain challenges still remain such as off-target systemic toxicity and multi-drug resistance. To circumvent these challenges, nanomedicine has been certainly beneficial as it helps in the efficient delivery of the chemotherapeutics to the target tissue^2^. There are currently around 50 FDA approved cancer nanomedicines available in the market, illustrating the primary focus of nanomedicines has been on cancer treatment in the recent past 3. The advent of smart nanocarriers having the potential to deliver hydrophobic and hydrophilic pharmaceuticals in a controlled manner has mitigated the cancer treatment scenario^4^, ^5^. Nanocarriers can substantially make a difference in anticancer treatment by altering the pharmacokinetics and enhancing the tissue distribution of the drug. Nanomaterials accumulate at the tumor site either by passive or active targeting. Active targeting is accomplished by attuning the nanocarrier surfaces via specific ligands so that they can bind to these highly expressed receptors on cancer cells. On the contrary, passive targeting relies on features of the tumor tissue like aberrant basement membranes, expanding endothelial cells, and even the absence of pericytes resulting in dilated vasculature which ultimately aids in enhanced uptake of the nanoparticles. Inflammation or hypoxia, which is typical for tumors, leads to thinning of the endothelium of blood vessels making the nascent blood vessels more permeable than they would have been in their normal state. Such blood vessels selectively permit the nanoparticles uptake into the tumor stroma. Also, the deficit of normal lymphatic drainage in the tumor region augments the retention of NPs^6^.

The enhanced permeability and retention (EPR) effect first emanated in the 1980s, and numerous efforts were made to comprehend the significance of this phenomenon in cancer targeting and to develop suitable treatments^6^. Several parameters, including tumour vascular permeability, the extracellular matrix, intratumoral pressure and regional blood flow, have been associated to the EPR effect^7^. EPR effect is also heavily reliant on nanoparticles characteristics such as its particle size (preferably between 20 to 200nm), surface chemistry as well as charge^6^. The EPR effect occurs in different types of tumours, and does not depend on cancer cell surface receptors or markers that vary across tumours. This broad applicability streamlines targeting and facilitates a pragmatic approach against cancer via EPR-based drug delivery. The EPR effect relies on the passive accumulation within tumour tissues avoiding the need for targeting ligands or receptor-specific interactions; simplifying the design and development of drug delivery systems. EPR effect is more pronounced in tumour tissues compared to healthy tissues, allowing preferential accumulation of therapeutic agents in tumours while minimizing exposure to normal tissues^8^.

Nanomedicine is typically applied for cancer therapeutics through one or a combination of treatment modalities, namely photothermal therapy, photodynamic therapy, chemotherapy, or immunotherapy^9^-^16^. Nanoparticles have been developed that utilizes near-infrared (NIR) responsive photo absorbers to produce local heat, i.e., photothermal transduction process to kill the cancer cells/tumor tissue upon NIR irradiation^17^. Nanoparticles (NPs) based on the photothermal transduction process is dependent on the localized accumulation of the nanoparticles. The visualisation of these nanoparticles is crucial to understand the tissue uptake, precise location within the tumour microenvironment, so that the intensity and duration of NIR irradiation can be controlled spatially and temporally^18^. The precise imaging and excellent temporal resolution offered by photoluminescence (PL) or optical imaging makes it favorable for early disease detection and evaluation of therapeutic activity^18^, ^19^. Near-infrared (NIR) dyes are excellent candidates for imaging as they are fluorescent, photothermally active as well as exhibit photodynamic properties. These dyes, when showered with NIR light of a specific wavelength, reach an excited state, and dissipate energy in the form of higher wavelength light (fluorescence), heat (photothermal effect) or reactive singlet oxygen (photodynamic effect). Additionally, NIR dyes also offer excellent sensitivity as a result of incredibly low organic tissue autofluorescence and absorption within the NIR spectral band, diminishing background interference and enhancing tissue penetration^20^. Several NIR dyes such as IR780, ICG, KSQ-4-H, BODIPY etc., are being extensively used for imaging and photothermal therapy of tumours^21^. IR780, a heptamethine cyanine dye has been the prototypical NIR dye candidate in recent years, due to its remarkable *in vivo* fluorescence, photostability as well as its mitochondrial targeting ability^22^. In comparison with free IR780, nanoparticles encapsulating IR780 have been reported to be retained within the tumor over a much longer period owing to EPR effect, showing higher fluorescence imaging intensity upon NIR irradiation^23^. However, with a lipophilic group, IR780 iodide is exceedingly difficult to dissolve in water, limiting its clinical applications. In addition, the toxicity of IR780 iodide remains a challenge, due to its low maximal tolerated dose of 1.5 mg/kg in rodents^24^. Moreover, due its weak solubility in biological fluids, rapid clearance, and minimal tumor uptake, IR780 needs to be loaded/administered at a significantly higher concentration to obtain detectable fluorescence signal^25^.

Plasmon resonance-enhanced fluorescence or metal enhanced fluorescence can foster the NIR dye's fluorescence at low biocompatible concentrations. It is a phenomenon in which the presence of a metal dramatically increases the fluorescence of a fluorescent molecule/dye. The strong electric field enhances the dye's excitation rate and fluorescence, which accounts for the rise in emission intensity^26^-^30^. For most of the plasmon enhanced fluorescence applications, gold (Au) has been the most extensively used metal, owing to biocompatibility, ease of synthesis, and unique optoelectronic properties^31^. Excellent photothermal property of gold due to localized surface plasmon resonance can be leveraged to obtain maximum fluorescence from the NIR dye, for its bimodal imaging and photothermal-based applications.

In one of our previous works, we developed a novel nano lipo polymeric hybrid system (PDPC NPs), coated with gold forming PDPC-IR-Au NPs and reported increased intracellular ROS, DNA damage and apoptotic cell death in MCF-7 cells. We further loaded this nanosystem with an NIR dye, IR780 and coated with gold (PDPC-IR-Au NPs) and demonstrated plasmon resonance enhanced fluorescence for the first time in a nanosystem. Additionally, these nanoparticles exhibited remarkable photothermal killing of MCF-7 breast cancer cells *in vitro* as well as in murine xenograft model, when injected intra-tumorally^30^. In continuation with our previous work, in this paper we report the EPR based targeted imaging of gold coated IR780 loaded lipo-polymeric nanoparticles (PDPC-IR-Au NPs, would now be referred as PIR-Au NPs), in metastatic breast cancer. The plasmon-enhanced fluorescence of PIR-Au NPs was utilised for the imaging of tumor cells and zebra fish embryos. Plasmon resonance enhanced fluorescence and EPR effect of PIR-Au NPs was investigated through *in vivo* imaging of animals. The photothermal heat generated *in-vivo* by EPR effect was also evaluated. We believe this novel nanosystem could be a promising tool for metal enhanced fluorescence guided imaging and as a potential therapy for tumors in future.

## Materials and methods

The current study has the same materials and methodology as reported in our previous work, with few modifications^30^. The detailed materials and methods section has been included in the supplementary information. The lipo-polymeric hybrid nanoparticles are referred to as PDPC NPs, IR780 loaded PDPC nanoparticles as P-IR NPs and Gold coated IR780 loaded PDPC nanoparticles as PIR-Au NPs throughout this study.

## Results and Discussion

The lipo-polymeric hybrid nanoparticles (PDPC NPs) were synthesized by a modified hydrogel-isolation technique as reported in our earlier publication^30^. An infrared dye, IR780 is a hydrophobic dye, known for its fluorescence could be used to track nanoparticles *in-vitro* and *in-vivo*^32^-^35^.

**Figure 1:**
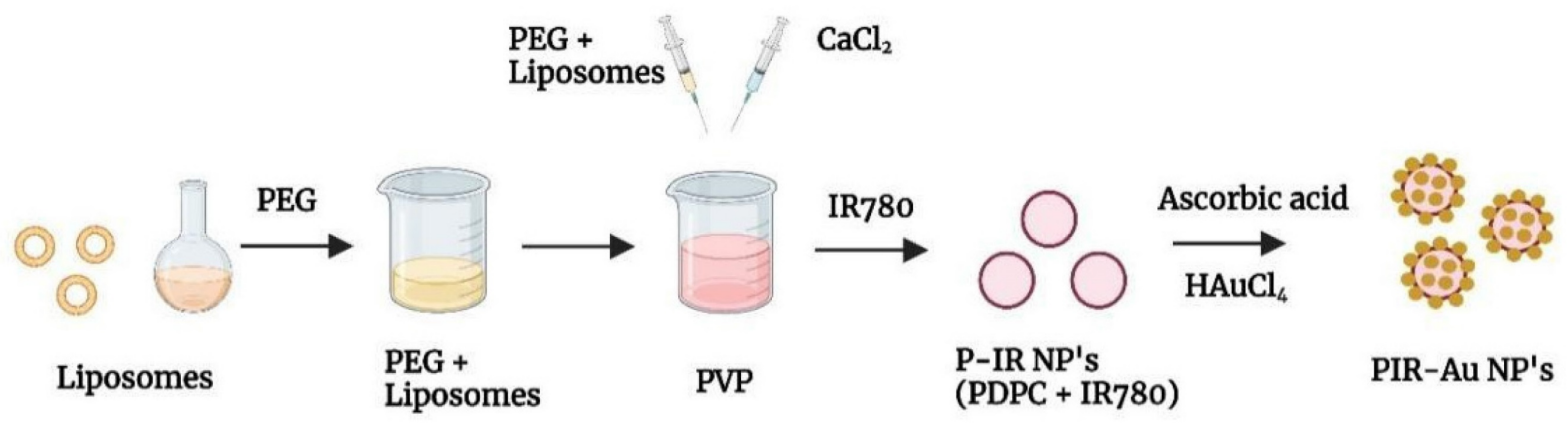
Schematics showing synthesis of PIR-Au NPs.

**Figure 2:**
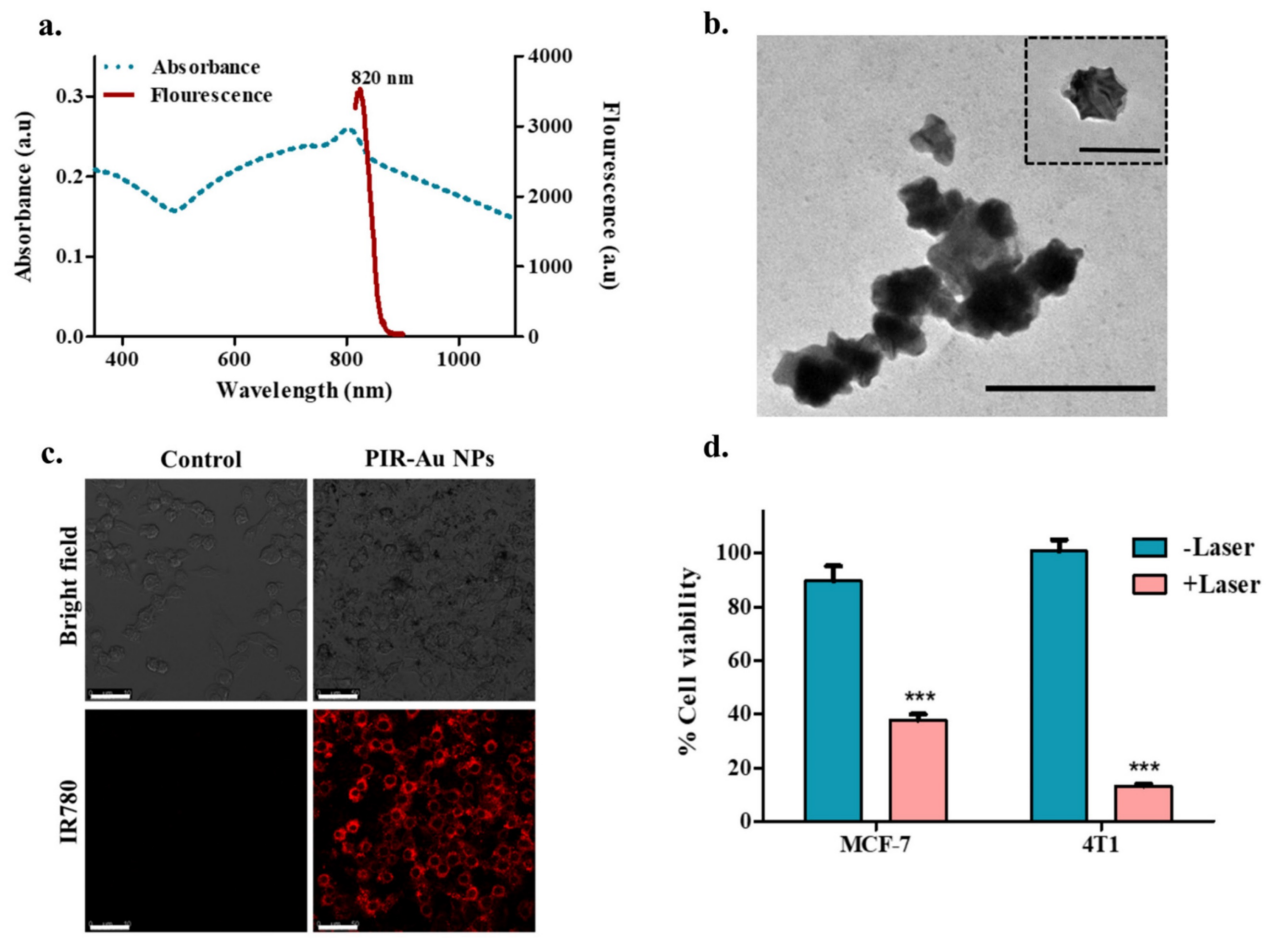
** Characterization and *in-vitro* assessment of PIR-Au NPs:** a) Spectral (absorbance and fluorescence) analysis and b) TEM imaging of PIR-AuNPs (Inset shows a single nanoparticle; *Scale bar corresponds to 200nm), c) Cellular uptake of PIR-AuNPs (*Scale bar corresponds to 50µm), d) *In-vitro* photothermal mediated cytotoxicity of P-IR-Au in breast cancer cell lines (MCF-7 and 4T1).

We reported a successful encapsulation of this dye in our nanosystem using the modified hydrogel isolation technique as shown in **Figure 1** and have demonstrated an increase in fluorescence with surface coating of gold, owing to the plasmon resonance fluorescence or metal enhanced fluorescence in our earlier reports^30^. The successful encapsulation of IR780 could be corroborated from the spectral studies, with the nanosystem showing an absorbance and fluorescence in the NIR region as shown in **Figure 2a & S1**. IR780 dye has a specific absorbance at 780nm, which red-shifts to 800 nm upon its encapsulation into a hydrophilic nanosystem^35^, ^36^. On further coating the surface of the lipo-polymeric hybrid system with gold (Au), by a simple reduction using ascorbic acid, PIR-Au NPs are formed. The near-infrared absorbance (600-900nm) with a specific absorption peak around 800nm and the fluorescence around 820nm indicates the successful encapsulation of IR780 in the lipo-polymeric hybrid nanosystem.

The fluorescence intensity of IR780 loaded PDPC NPs (P-IR NPs) significantly increased with Au surface coating (PIR-Au NPs), which could be observed from **Figure S2**. The increased fluorescence could be credited to plasmon enhanced fluorescence or metal enhanced fluorescence, which was specific to this lipo-polymeric hybrid nanosystem^30^.

The TEM imaging of PIR-Au NPs in **Figure 2b** shows the surface coating with gold. The uptake of PIR-Au NPs has been assessed in mice breast cancer cells (4T1). **Figure 2c** shows an enhanced uptake of PIR-Au NPs as compared to free IR780, while maintaining the concentration of IR780 and incubation time constant. This shows the improved bioavailability of IR780 when loaded into the nanosystem, as compared to free IR780. The cellular uptake of free IR780, P-IR NPs and PIR-Au NPs are shown in the **Figure S3**. These nanoparticles were photo thermally active with a near infrared absorbance (**Figure 2a**). Hence, we evaluated the photothermal mediated cytotoxicity in two different breast cancer cell types i.e., MCF-7 (human origin) and 4T1 (mouse origin). A significant cell death is noted in the breast cancer cells, when treated with PIR-Au NPs followed by NIR laser irradiation (808nm), as can be seen in **Figure 2d.**

**Figure 3:**
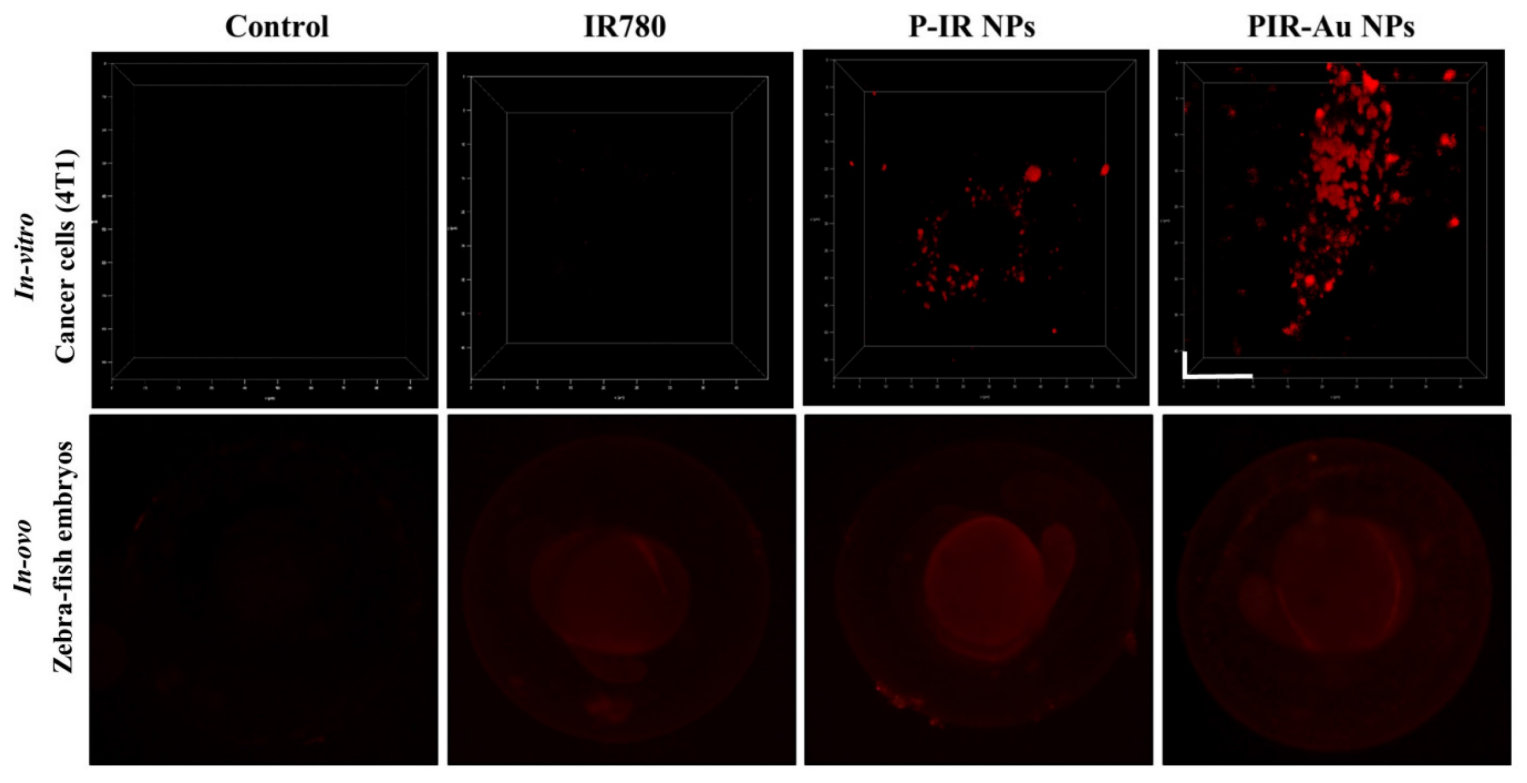
** Plasmon resonance enhanced fluorescence of PIR-Au NPs** a)* in-vitro* (4T1 breast cancer cells, incubated for 6h, washed, and fixed in formaldehyde) (*scale bar corresponds to 10*10µm) and b) *in-Ovo* (zebrafish embryos incubated with nanoparticles and free dye for 24 h and imaged at 4x magnification).

We further evaluated these nanoparticles *in-vitro* (in 4T1 cells) and in zebra fish embryos to understand their uptake and increase in fluorescence as compared to free IR780 and the lipo-polymeric hybrid system just loaded with IR780, i.e., P-IR NPs. The three-dimensional view of cells in **Figure 3a** shows the enhanced fluorescence of PIR-Au NPs, while, the free IR780 dye showed no fluorescent signal and the P-IR NPs showed slight fluorescence, at the same concentrations of IR780. The uptake in zebrafish embryos also depicts an enhanced fluorescence in the groups incubated with PIR-Au NPs, as compared to free IR780 or P-IR NPs (**Figure 3b & S4**). The fluorescence of PIR-Au NPs could be seen all over the embryo including the sac, following 24 h incubation with the nanoparticles. A higher intensity in fluorescence was recorded with a longer incubation time i.e., 48 h^30^.

**Figure 4:**
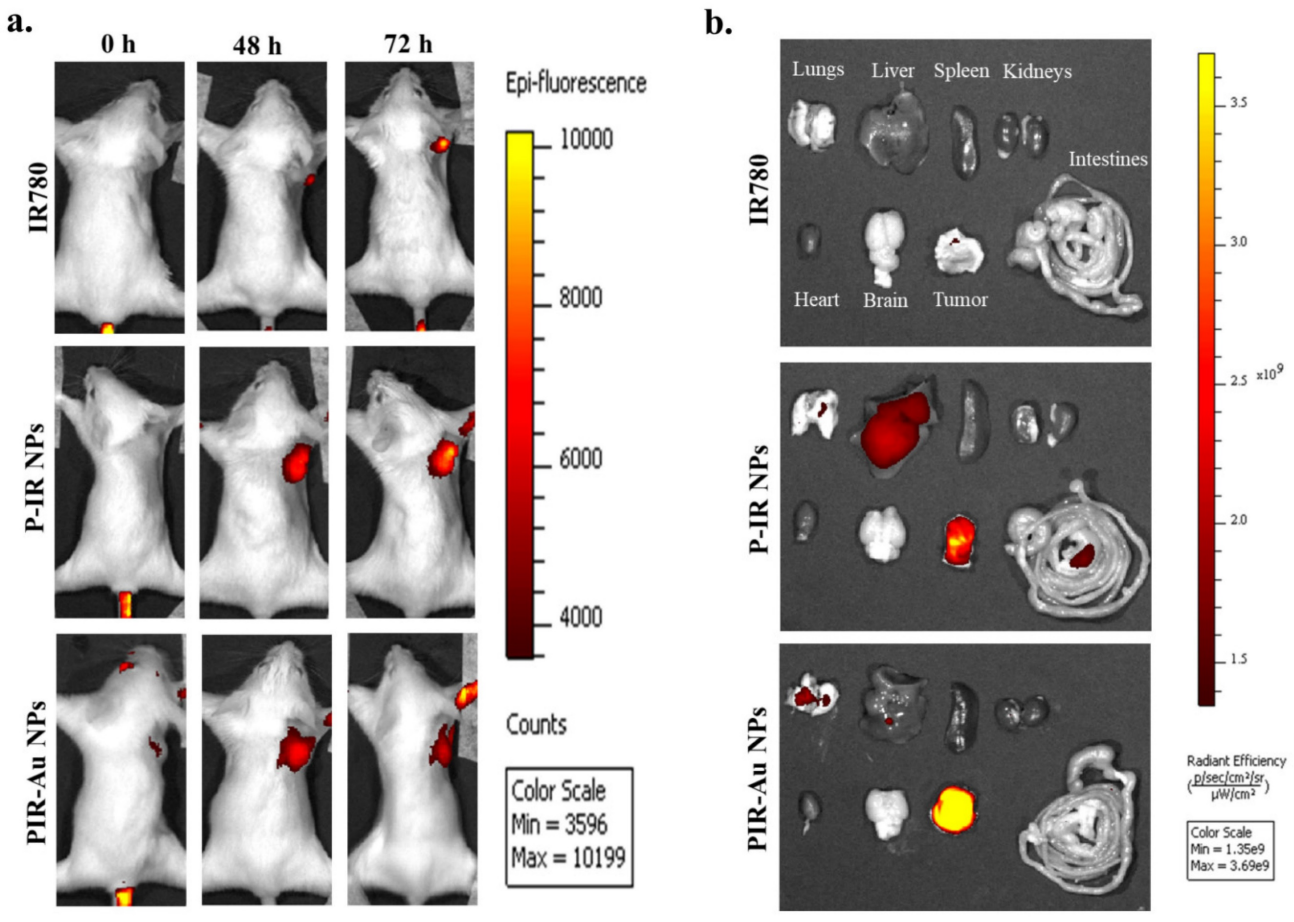
** Plasmon resonance enhanced fluorescence and EPR of PIR-Au NPs:** a) IVIS imaging presenting *in-vivo* tumor accumulation (4T1 breast cancer model), b) *Ex-vivo* fluorescence from collected organs.

With these observations* in-vitro* and *in-ovo*, we have analyzed the efficacy of the nanosystem *in-vivo* in a breast cancer tumor model developed in Balb/c mice. The enhanced permeation and retention effect was noted with PIR-Au NPs. **Figure 4(a & b)** and **Figure S5** shows the EPR effect of PIR-Au NPs. The nanoparticles (P-IR NPs and PIR-Au NPs) and free IR780 were injected intravenously at the same concentrations of IR780 and were monitored using *in-vivo* imaging system. The fluorescence was first recorded at the tumor site within 9 h of injection, with an increase in intensity up to 72 h. After 72 h, no significant increase in the fluorescence intensity was noted; the mice were sacrificed and the organs were collected from the three groups. A strong fluorescent signal was recorded at the tumor site from mice administered with both P-IR and PIR-Au NPs for 72 hours. However, the tumor tissues collected after 72 h, clearly showed a greater fluorescence with PIR-Au NPs, as compared to P-IR NPs or IR780 only. The P-IR NPs also seem to have accumulated in the liver, while the PIR-Au NPs showed no such accumulation. In addition, PIR-Au NPs treated mice showed a fluorescent signal in the lungs tissue, indicating their accumulation in lungs, which could be employed for treating lung metastasis, if any. IR780 dye has been known for its EPR effect, i.e., passive targeting by accumulating in the tumor site^33^-^37^. A number of nanosystems loaded or conjugated with IR780 have shown similar effect^38^, ^39^ but at very high concentrations of IR780. However, in this report, we show the EPR effect at very less concentrations of IR780, owing to the plasmon resonance enhanced fluorescence or metal enhanced flourescence. PIR-Au NPs showed a significant fluorescence signal at very less concentration of IR780, without compromising EPR effect which could help in excellent imaging. The smaller concentrations of IR780 dye with the enhanced fluorescence by the surface coating of Au not only overcomes the issues of toxicity, but also provides insights into metal enhanced fluorescence. This becomes an advantage due to the detectable fluorescence signal and a prominent EPR effect without the need for higher concentrations of IR780. The nanosystem imparts the hydrophilic nature to the IR780 dye and the surface coating with gold further enhances the fluorescent signal by plasmon-enhanced fluorescence^33^, overcoming the limitations of solubility and toxicity of IR780.

**Figure 5:**
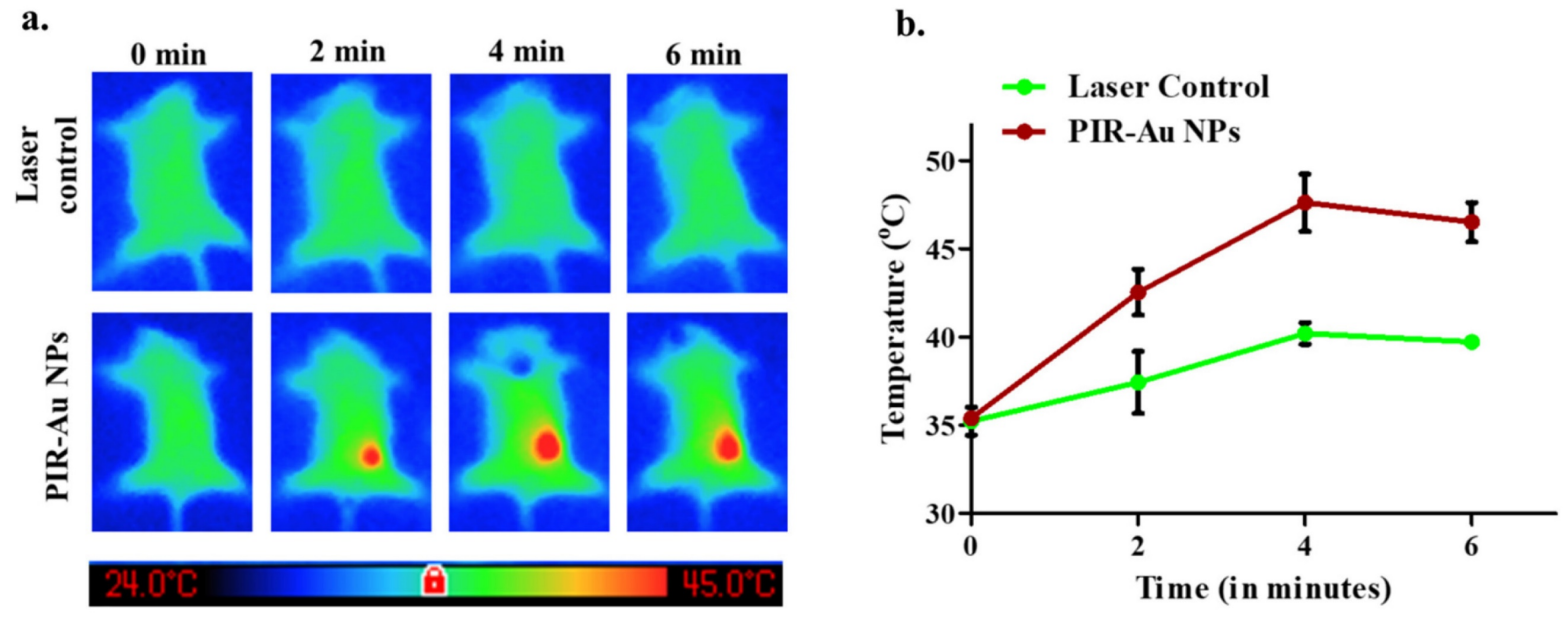
** Photothermal heat *in-vivo* by EPR effect:** Thermal imaging showing the heat generated a) 24 h post intravenous injection of PIR-Au NPs in orthotopic 4T1 model, b) Temperature increment corresponding to the laser irradiation time *in-vivo.*

We have earlier reported the photothermal therapeutic potential of PIR-Au NPs in mice, when injected intra-tumorally^33^. With a significant accumulation of PIR-Au NPs by EPR effect at the tumor site, we have evaluated the photo thermal transduction efficiency of the PIR-Au NPs *in-vivo* (**Figure 5a** & **Figure S6)**. The PIR-Au NPs were injected intravenously and 24 h post the treatment, the tumors were subjected to NIR laser (808nm) irradiation. The rise in temperature was measured across the two groups: PIR-Au NPs & laser control. Mice with only laser irradiation at the tumor site served as laser control. A clear and a gradual increase in temperature was recorded in PIR-Au NPs treated group, when juxtaposed to the laser control group (**Figure 5b**). This rise in temperature could be used effectively for photothermal-based imaging process. These results indicate the potential of this nanosystem for its passive targeting. In addition, if the nanosystem is further loaded with anti-cancer agents, it would be more beneficial, with the photothermal heat acting as a stimulus for the drug release. This lipo-polymeric hybrid nanosystem could therefore be successfully applied for image-guided, passive targeted therapy to achieve synergistic therapeutic benefits.

## Conclusion

This article describes the EPR-based targeting and imaging with a novel lipo-polymeric nanosystem, PIR-Au NPs. Spectral studies confirmed the successful encapsulation of an NIR dye IR780 into the nanosystem and TEM imaging confirmed the surface coating with gold. Enhanced uptake of PIR-Au NPs, recorded by a strong fluorescent signal was observed in murine mammary carcinoma cell line 4T1 compared to unbound IR780 at the identical concentrations and period of incubation, depicting the improved bioavailability of IR780. The photothermal mediated cytotoxicity in two distinct breast cancer cell lines, MCF-7 (human origin) and 4T1 (mouse origin), revealed significant cell mortality. The nanosystem exhibited an increase in fluorescence due to the metal-enhanced fluorescence phenomenon. An enhanced fluorescence was observed both *in-vitro* and *in-ovo* with PIR-Au NPs, as compared to free IR780 or P-IR NPs, highlighting the plasmon resonance enhanced fluorescence with the gold coating. PIR-Au NPs also showed enhanced permeation and retention effect *in-vivo* in a breast cancer model. PIR-Au NPs showed significant tumor accumulation at very less concentrations of IR780 without compromising the EPR-based passive accumulation or fluorescence signal. The reduced concentrations of IR780 dye with the fluorescence augmented by the metal coating of Au not only tackles the toxicity issues, but also provides insights into metal-enhanced fluorescence, which is highly beneficial, offering excellent detectable fluorescent signals not at the expense of large quantities of IR780.

In addition, we also showed the photothermal performance of PIR-Au NPs *in vivo*. Compared to the laser control group, mice administered with PIR-Au NPs exhibited a significant increase in surface temperature at the site of tumor, with the temperature increasing progressively. The preceding temperature increment results indicate that this nanosystem has the potential for EPR-based targeting at the tumor site. In addition, the nanosystem is expected to be even more superior if it were loaded with anti-cancer agents, with photothermal heat functioning as a stimulus for drug release, a synergistic effect could be achieved. In addition to loading of drugs in the nanosystem, the therapeutic effect of the accumulated nanoparticles at the targeted site needs to be thoroughly investigated. Therefore, this lipo-polymeric hybrid nanosystem could be effectively applied as an image-guided, passive targeting vehicle with enhanced photothermal transduction property.

## Supplementary Material

Supplementary materials and methods, figures.
