# Supplementary material for: A lipo-polymeric hybrid nanosystem with metal enhanced fluorescence for targeted imaging of metastatic breast cancer
Source: Nanotheranostics. 2024 Feb 17;8(2):239–46. doi: 10.7150/ntno.92410 (PMC10911974; doi:10.7150/ntno.92410)
Supplement: Supplementary file 1 — Supplementary materials and methods, figures. [file ntnov08p0239s1.pdf]

A lipo-polymeric hybrid nanosystem with metal enhanced fluorescence for targeted imaging of metastatic breast cancer

TejaswiniAppidi[a#], RajalakshmiP.S[a#], Shubham A. Chinchulkar[a], Paloma Patra[a], Kavipriya Murugaiyan[a],Bantal Veeresh[b], Aravind Kumar Rengan*[a]

[a] Dept. of Biomedical Engineering, Indian Institute of Technology Hyderabad, India.

[b] G. Pulla Reddy College of Pharmacy, Hyderabad, India

* Corresponding Author email: aravind@bme.iith.ac.in

# Equal Contribution

Supplementary information

Materials and Methods

Materials

The lipid 1,2-Dioleoyl-sn-glycero-3-phosphoserine, sodium salt (DOPS-Na) was obtained as a generous gift from Lipoid, Germany. Poly ethylene glycol (Mw: 6000), Calcium Chloride anhydrous ($CaCl_2$), Sodium Chloride, Ascorbic acid, MTT (3- (4,5- Dimethylthiazol -2 -yl) - 2,5 Diphenyltetrazolium Bromide) were procured from SRL Chemicals, India. Polyvinylpyrrolidone (PVP, K-15) was obtained from Merck, Millipore. IR-780 iodide, Hydrogen Tetrachloroaurate (III)/Chloroauric acid ($HAuCl_4.3H_2O$), Solvents: Methanol, Chloroform, and Dimethyl sulfoxide (DMSO) were procured from SRL chemicals, India. Phosphate buffer pH 7.0, Dulbecco's Modified Eagle Medium (DMEM), Roswell Park Memorial Institute Medium (RPMI-1640), and Calf Serum (US origin), were purchased from Himedia Chemicals, (Mumbai, India). All the chemicals were used as received without any further purification. Female Balb/C mice (4-6 weeks) procured from Hylasco Bio-Technology Pvt. Ltd, Hyderabad. The animals were kept in normal condition to allow acclimatization in environmental conditions.

Methods

Characterization

The absorption spectra were recorded by a UV-Visible spectrophotometer (UV-1800, Shimadzu, Japan). The fluorescence measurements were recorded by an Enspire multimode plate reader (Perkin Elmer, USA) and Fluorescence spectrophotometer (RF 6000, Shimadzu, Japan). The size and morphology of the nanoparticles were characterized by JEOL, Transmission Electron Microscopy (TEM) (JEOL, JEM 2100, JEM-2100F, USA). The imaging of zebra-fish embryos was performed using a fluorescent microscope (Olympus, CKX-53, USA) and a confocal scanning laser microscope (CSLM, Leica TCS SP8, Germany). The

thermal images were captured using FLIR thermal camera (Chauvin Arnoux, CA, 1950 IR camera). 808 nm NIR laser (808 nm, 650 mW, Shanghai Inter-Diff Optoelectronics Technology Ltd, Shanghai, China) was used for all the Photothermal experiments. The phantom imaging was performed by the *In-vivo* Imaging System (IVIS) Lumina fluorescence imaging system (Perkin Elmer, USA).

Cell lines & Maintenance

The human breast carcinoma (MCF-7) cell line was obtained from the National Centre for Cell Sciences (NCCS), Pune, India. The murine breast carcinoma (4T1) cell line was obtained from AddexBio (C0006004), San Diego. The MCF-7 and 4T1 cells were cultured and maintained using DMEM and RPMI-1640 medium respectively, supplemented with 10% (v/v) Fetal bovine serum (FBS), and 1% penicillin-streptomycin solution and were maintained at 37°C in a humidified atmosphere, containing 5% CO_2 under sterile conditions [30].

Zebrafish Husbandry

Zebrafish (Danio rerio) was obtained as a generous gift from CSIR-Centre for Cellular and Molecular Biology (CCMB), Hyderabad, India. Zebrafish were raised and maintained in a closed flow-through culture system at 28 ± 0.5°C in a 14:10 h light−dark cycle. The zebrafish were fed twice a day. In the following light cycle, embryos were collected 2h postfertilization and washed several times with standard E3 culture media and healthy embryos at the blastula stage were selected for imaging [32,33].

In-vitro studies

P-IR-Au NPs for imaging of mouse breast cancer cells and zebrafish embryos

Imaging of 4T1 cells

The 4T1 cells were imaged using confocal scanning microscope with the study protocol mentioned as follows. The 4T1 cells were seeded at a density of $1x10^5$ cells/well on a coverslip in a 6 well plate. Following their incubation, the cells were treated with free IR780, P-IR NPs and P-IR-Au NPs diluted in fresh medium. The concentration of IR780 maintained constant at 2 µg in all three groups. Untreated cells were considered as control. The cells were incubated for 6h before being rinsed three times with PBS and fixed with 4% formaldehyde. Finally, confocal scanning laser microscopy was used to image the cells, with an excitation laser of 633 nm and an emission channel in the range of 600 to 800 nm[32,34,35]. A 3D imaging view was recorded in confocal microscopy by magnifying a single cell.

Imaging in Zebrafish embryos

At 2 h after fertilisation (hpf), zebrafish embryos were collected and cleaned three times using a regular E3 culture medium. In a six-well plate, the embryos were distributed 10 per well in 2 mL of media. The treatment groups for the embryos included P-IR-Au NPs, P-IR NPs, and free IR780, with a constant concentration of IR780 (0.5 µg) in each group. The untreated embryos served as the control group. Following an incubation for 24 h, the embryos were washed and then imaged under fluorescence microscope (Olympus CKX-53 (USA)) [33,36].

Photothermal Mediated Cytotoxicity

The photothermal mediated cytotoxicity was investigated in both the breast cancer cell lines i.e., MCF-7 and 4T1 cells by MTT assay. The cells were seeded at a cell density of $1x10^4$ cells/well. Post 24 h, P-IR-Au NPs diluted in cell culture media were added to the wells and exposed to 808 nm NIR irradiation for 5 min. Cell viability was assessed using the methyl thiazolyl tetrazolium (MTT) after 24 h of irradiation. Cells without any treatment and cells treated with P-IR-Au NPs/NIR laser were considered controls[32,34,35].

In-vivo studies

Development of 4T1 breast cancer model in mice for evaluation of EPR effect

All the *in-vivo* experiments were carried out according to the CPCSEA guidance and Institutional Animal Ethics Committee of G. Pulla Reddy College of Pharmacy, Hyderabad (GPRCP/IAEC- 02/29/12/2021/PCL-14). The female Balb/C mice (4-6 weeks), used in study were procured from Hylasco Bio-Technology Pvt. Ltd, Hyderabad. The tumor model was established by injecting approximately 1×10^6 4T1 cells/animal subcutaneously to the dorsal flank region of female Balb/C mice. When the tumor reached ~150-200 mm^3, the animals were randomly grouped into three groups: first group received IR780, second group received P-IR NPs and third group received P-IR-Au NPs via tail vein i.e., intravenously at the concentrations of IR780: ~10 µg and gold: ~196 µg. The mice were imaged at 0, 9, 12, 24, 48 and 72 h, following the injection of the nanoparticles. The mice were anesthetized and distribution of different formulations were visualized or examined by using *In-vivo* Imaging System (IVIS) Lumina fluorescence imaging system (Perkin Elmer, USA). After 72 h, mice were sacrificed, tumors and organs were collected and *ex-vivo* fluorescence images were taken using IVIS[30,32,34,37,38].

Photothermal transduction effect of P-IR-Au NPs *in-vivo*

The photothermal transduction effect of the P-IR-Au NPs was evaluated as follows. This was performed in two different models. Mice with subcutaneous tumors and orthotopic tumors. The P-IR-Au NPs were administered into mice intravenously and 24 h post-injection, the mice were irradiated using 808 nm NIR laser at tumor site in various directions for total 6 min. The temperature was recorded at 0, 2, 4, and 6 min and the thermal images were recorded using thermal imaging camera (Chauvin Arnoux, CA,1950 IR camera, USA)[37,38].

Statistical analysis

Mean ± SEM values were used for the expression of data. Statistical analyses of data were performed using the student's t-test or two-way ANOVA. Values of $p < 0.05$ were considered statistically significant. (The statistical significance was denoted as follows: for $p > 0.05$: nonsignificant (ns), for $p < 0.05$: *, for $p < 0.001$: ***).

<u>Supplementary Figures</u>

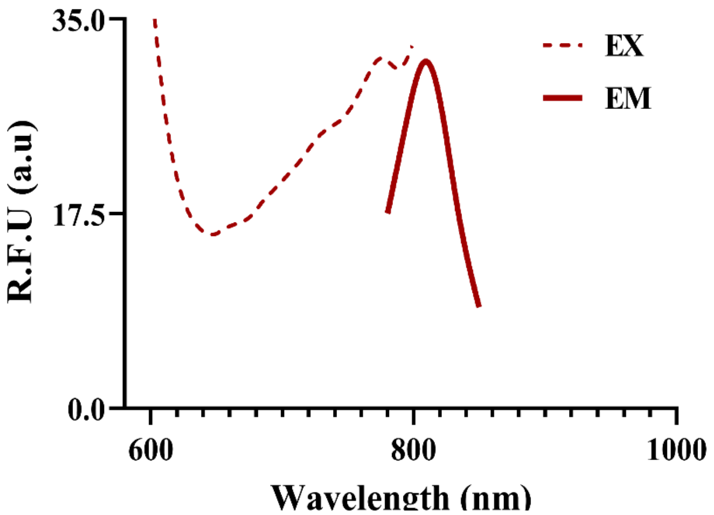

Figure S1: Excitation and Emission of IR780

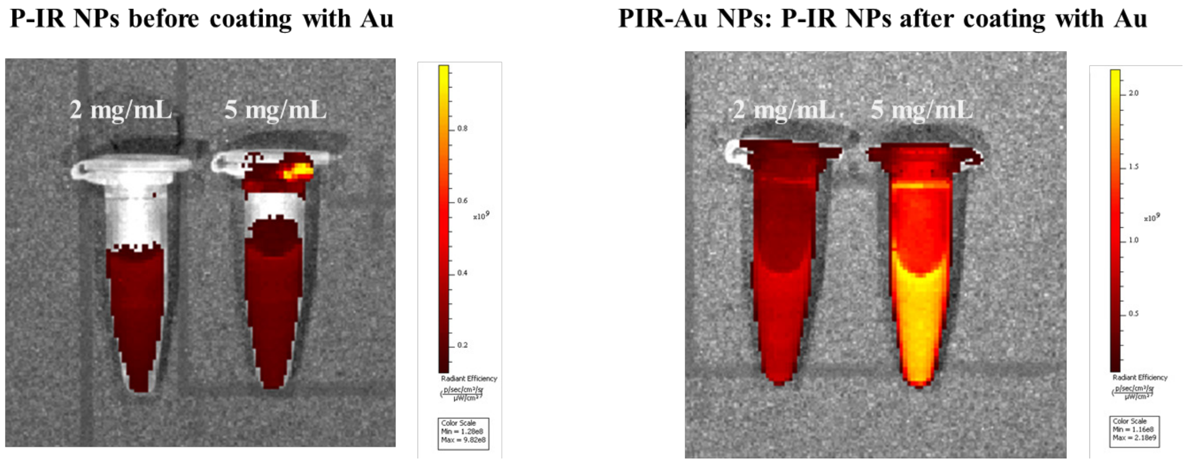

Figure S2: *In-vivo* phantom imaging showing the enhanced Plasmon resonance fluorescence of P-IR NPs with surface coating of gold.

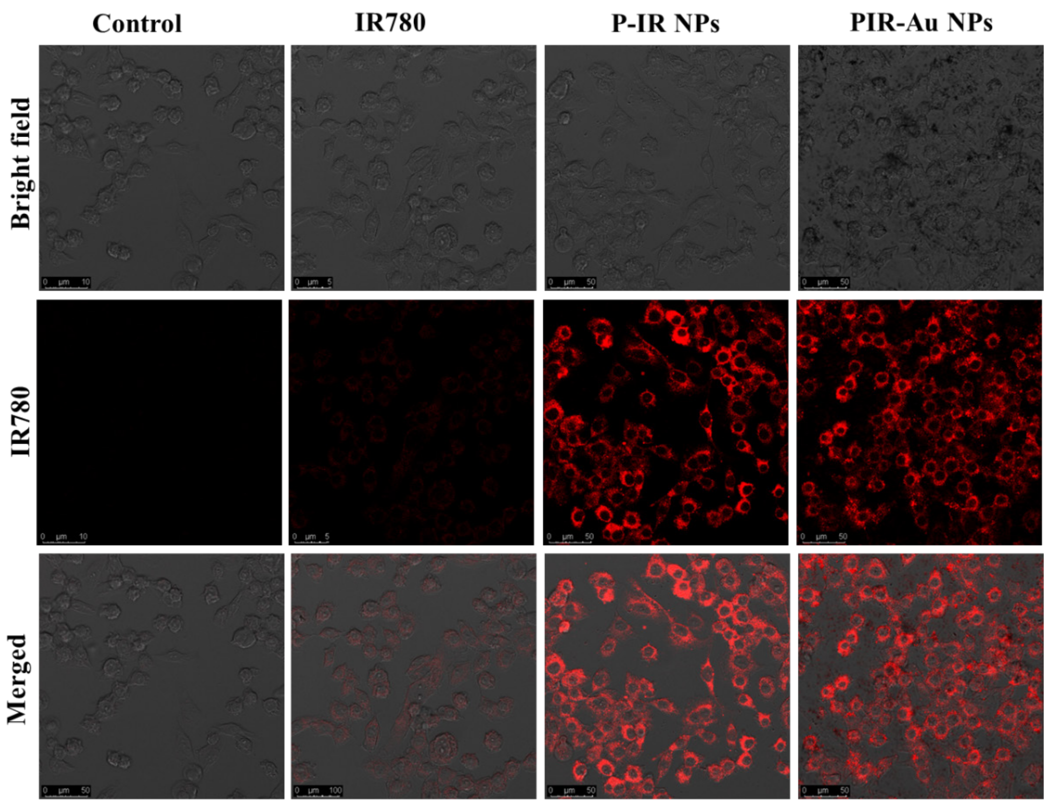

Figure S3: Cellular uptake of IR80, P-IR NPs and PIR-Au NPs in 4T1 cells (*Scale bar corresponds to 50µm).

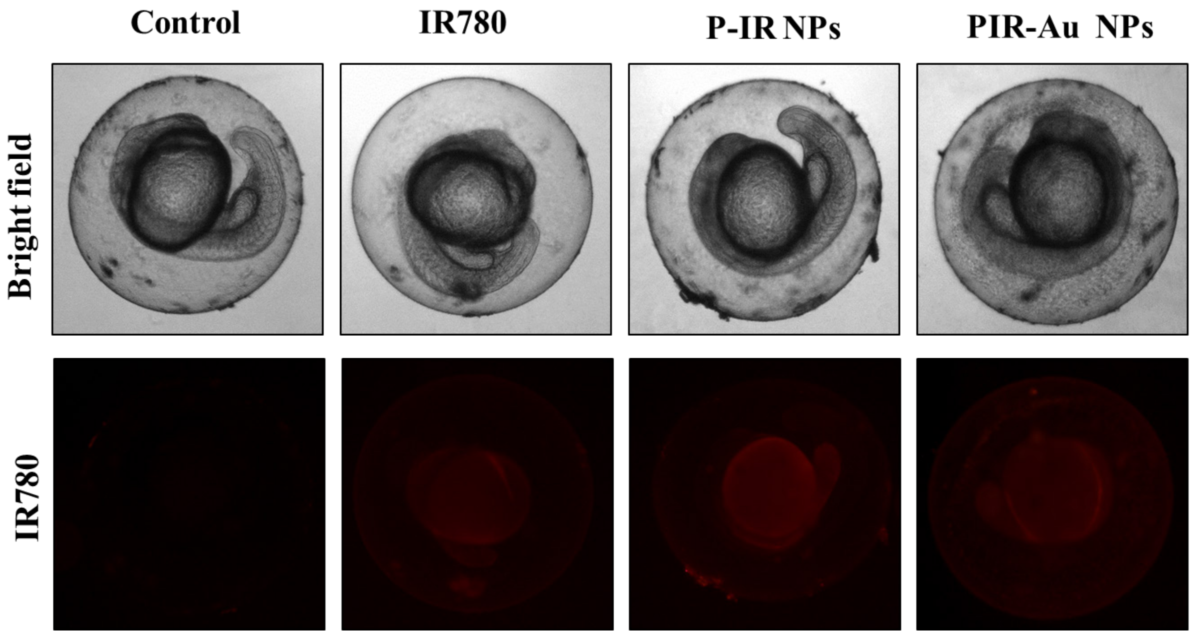

Figure S4: Plasmon resonance enhanced fluorescence in Zebrafish embryos (Imaged under 4x magnification).

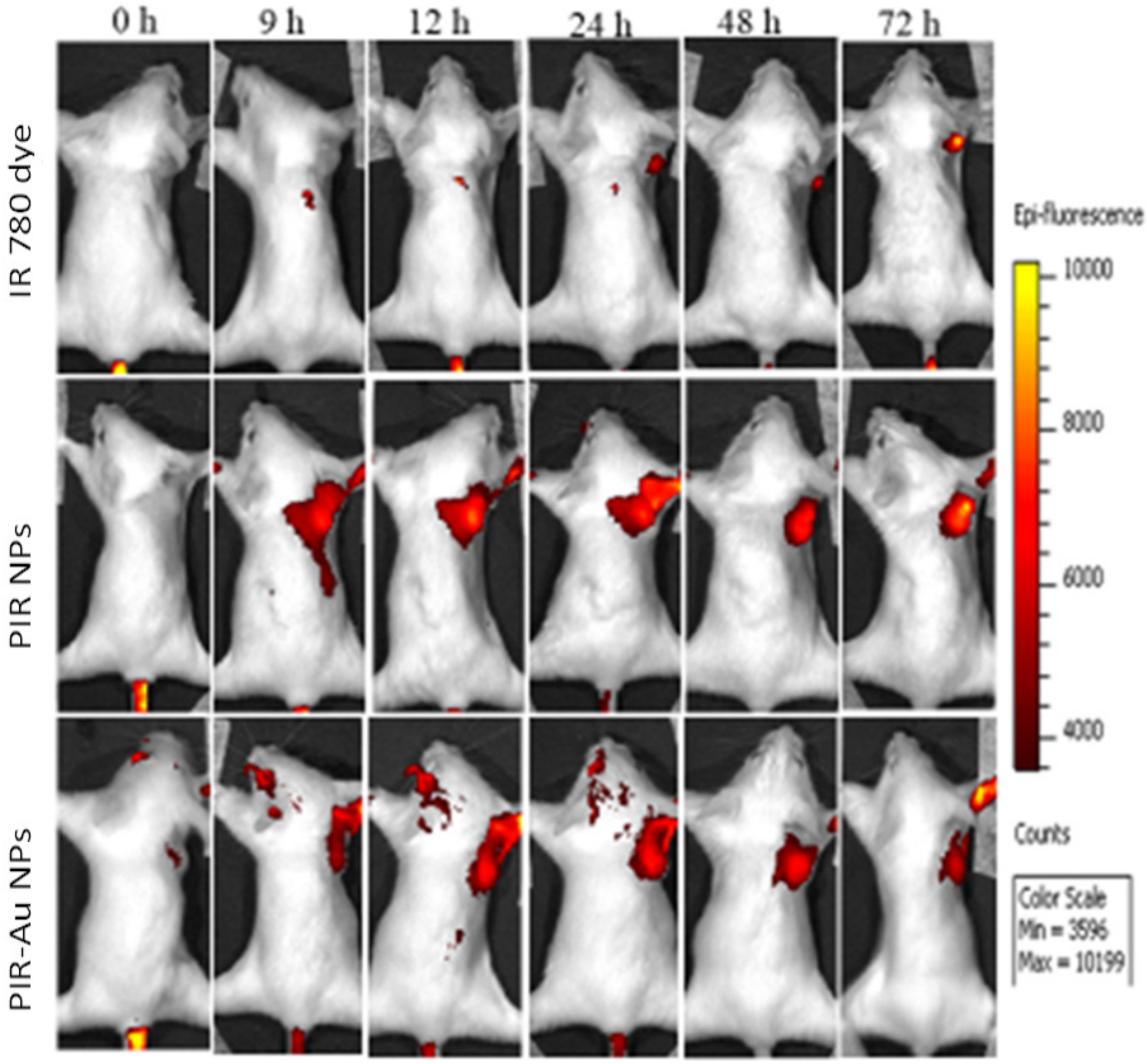

Figure S5: IVIS imaging showing the tumor accumulation of intravenously injected PIR-Au NPs over a period of time (0-72 hours).

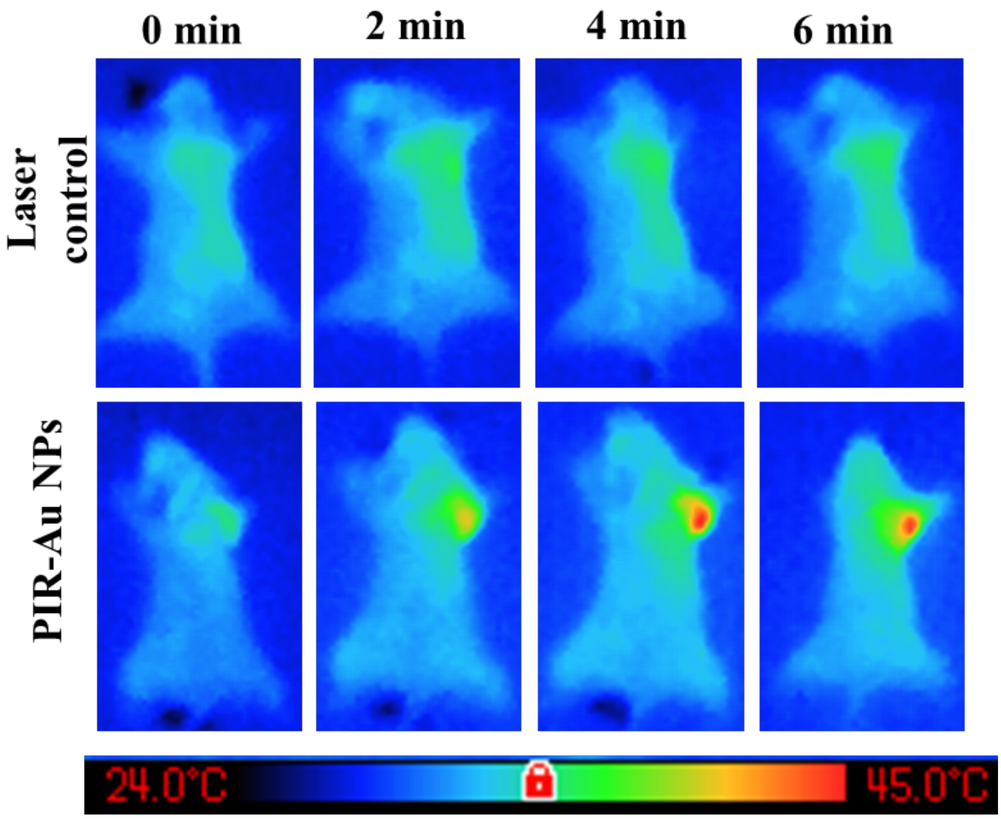

Figure S6: Thermal imaging showing the photothermal heat generated 24 hours post intravenous injection of PIR-Au NPs in subcutaneous 4T1 model.
